# Accelerometry cut points for physical activity in underserved African Americans

**DOI:** 10.1186/1479-5868-9-73

**Published:** 2012-06-14

**Authors:** Nevelyn N Trumpeter, Hannah G Lawman, Dawn K Wilson, Russell R Pate, M Lee Van Horn, Alicia K Tate

**Affiliations:** 1 Department of Psychology, University of South Carolina, Barnwell College, Columbia, SC 29208, USA; 2 Arnold School of Public Health, University of South Carolina, Columbia, SC 29208, USA

**Keywords:** Actical, Older adults, Low-income adults, African american adults, Calibration, Moderate physical activity, Cut points

## Abstract

**Background:**

Despite their increased use, no studies have examined the validity of Actical accelerometry cut points for moderate physical activity (PA) in underserved (low-income, high-crime), minority populations. The high rates of chronic disease and physical inactivity in these populations likely impact the measurement of PA. There is growing concern that traditionally defined cut points may be too high for older or inactive adults. The present study aimed to determine the self-selected pace associated with instructions to “walk for exercise” and the corresponding accelerometry estimates (e.g., Actical counts/minute) for underserved, African American adults.

**Method:**

Fifty one participants (61% women) had a mean age of 60.1 (SD = 9.9) and a mean body mass index of 30.5 kg/m^2^ (SD = 6.0). They performed one seated task, one standing task, and three walking tasks: “strolling”; “walking for exercise”; and “walking in an emergency.”

**Results:**

The average pace for strolling, walking for exercise, and walking in an emergency were 1.62 miles per hour (mph; SD = .51), 2.51 mph (SD = .53), and 2.86 mph (SD = .58), respectively. The average Actical counts/minute for the five activities were: 4 (SD = 15), 16 (SD = 29), 751 (SD = 591), 2006 (SD = 1095), and 2617 (SD = 1169), respectively. Regression analyses showed that the predicted counts/minute for a pace of 2.0 mph (which is used as the criterion for moderate exercise in this study) was 1075 counts/minute (SEM = 73).

**Conclusions:**

The cut point associated with subjectively determined moderate PA is similar to those previously published for older adults and extends the use of adjusted cut points to African American populations. These results indicate that accurate cut points can be obtained using this innovative methodology.

## Background

Despite evidence that regular physical activity (PA) is associated with improved fitness and reduced incidence of chronic disease, the majority of Americans do not meet national recommendations of 150 minutes of PA per week [1,2]. Research on psychosocial and environmental correlates of PA as well as interventions to increase PA rely on accurate measurements of free-living PA and energy expenditure (EE) to assess outcomes. Accelerometers have been used for this purpose because they offer a number of advantages. Not only do they estimate the duration, frequency, and intensity of PA, they also are non-intrusive, convenient, and more objective than self-report measures [3]. Accelerometers can provide estimates of EE and time spent in various PA intensities when validated and calibrated in the population of interest [4,5]. The Actical (Mini Miter, Bend, OR, USA) device has been increasingly favored because of its improved reliability over other devices [6] and its demonstrated validity in adults [7-9] especially when positioned on the hip [4,10,11]. Despite this, to date only three studies have provided intensity specific Actical count thresholds for hip-mounted devices for adults that enable the classification of measured activity as light, moderate or vigorous [10,12,13], with only two for Actical count data collected in 60-second epochs [10,13], and only one using a sample of older adults [13]. Therefore, the purpose of the present study was to extend the current literature by defining cut points for older, African American adults. Not only did this study sample participants from a previously underrepresented population, it also utilized a novel methodology, defining the cut point against subjectively defined moderate intensity PA, which may be more appropriate for this population.

As mentioned above, researchers have emphasized the importance of calibrating accelerometers with a sample representative of the population of interest and with activities common to the population of interest [5]. Although Hooker and colleagues filled a gap in the literature by validating the Actical for use in older adults [13], two characteristics of the research design potentially limit the generalizability of the results. First, closely following Heil’s study design [10], Hooker and colleagues used a protocol including researcher-imposed locomotive activities. Participants walked on a treadmill at 2.5 miles per hour (mph) and 3.5 mph. However, locomotive activities at the lower end of the intensity spectrum may be more common activity types in older adults [e.g.,[14]. Fitzsimmons and colleagues, for example, showed a decline in absolute walking speed with an increase in age. Older participants “chose to walk at the same percentage of maximal walking speeds as young participants”; however, older participants walked 20% more slowly and resulted in a significantly higher maximum oxygen uptake (VO_2max_) at each of the four paces, with the most significant increase for older participants evidenced in the directives for “brisk” and “fast” paces [14]. Typically, older participants who walked at their perceived “slow” rate experienced a “relative aerobic demand” parallel to the young participants who walked at their perceived “fast” rate (47% of VO_2max_) [14]. In general, studies with adults aged 63 or older have found that older adults tend to self-select paces ranging from 1.5-2.2, 2.0-2.9, and 2.7-3.6 mph when instructed to walk at a “slow”, “normal/comfortable/usual”, or “fast” pace, respectively [14-16]. Thus, the locomotive activities in the studies by both Heil and Hooker and colleagues may not be representative of those typical in older adults.

Second, like Heil, Hooker and colleagues used relative oxygen uptake and standard metabolic equivalents (METs; calculated as relative oxygen uptake/3.5) as the measure of EE [10,13]. There is growing concern, however, that standard EE estimates may underestimate the actual EE for underserved adults including older populations [11,17,18] and inactive adults [2,17,18]. Both the 2011 Compendium of Physical Activities and the 2008 US Physical Activity Guidelines (USPAG) recognize that the absolute intensity values of most activities may not apply to older adults or less fit adults [2,17]. The USPAG further suggests that individuals at lower fitness levels, including physically inactive adults and some older adults, define the intensity of activities using a subjective assessment of “relative intensity” [2]. Therefore, the use of standard MET levels (light <3.0, moderate 3.0-5.9, vigorous ≥6.0) to determine intensity specific cut points may not be appropriate for populations with older adults or less physically active adults. This would include low-income, minority populations, which are known to have higher prevalence of physical inactivity and chronic disease [19-22].

Thus, the current study sought to expand on the current literature by defining accelerometry cut points for an older, African American adult population and by defining moderate-intensity PA by the participants’ subjective assessments of intensity. Accordingly, the purpose of the present study was two-fold: to observe the full range of locomotive activity in an older, African American adult sample and to identify the Actical cut point corresponding to subjectively-defined, or “relative”, moderate intensity PA for this group. Participants were allowed to self-select the pace they commonly used when “walking for exercise”, thus enabling the researchers to identify the range of locomotive activity common in our target population. The walking pace that included the self-selected pace for “walking for exercise” for the majority of the sample was used as the criterion of relative moderate PA. The study was designed to determine the Actical cut point corresponding to the pace subjectively defined as “walking for exercise” and how that differed from those obtained using standard MET intensity thresholds.

## Methods

### Participants

Fifty-one African American adults, age 45 or older were recruited from churches, community centers, and senior centers in Columbia, SC. Inclusion criteria were: (a) African American ages 45 and older, (b) no medical condition or disorder that would limit participation in moderate-intensity exercise and (c) able to take a 30-minute brisk walk. Participants were also screened with the Physical Activity Readiness Questionnaire [23] to ensure their safety during the physical activity tasks. The study protocol was approved by the University of South Carolina institutional review board. All participants provided written informed consent.

## Measures

### Demographics and anthropometrics

Age, gender, and other demographic information were provided by the participant with a self-administered questionnaire. Height and weight were measured by certified measurement staff following a standardized protocol. Height was measured to the nearest 0.1 centimeter (cm) with a portable stadiometer (Shorr Productions, Olney, MD). Weight was measured to the nearest 0.1 kilogram (kg) using an electronic scale (SECA, Model 880, Hamburg, Germany). The average of two measurements was used for both height and weight.

### Accelerometry

Acticals are omni-directional accelerometers used to estimate PA duration and intensity. The Acticals were initialized with a 60 second epoch length and, thus, recorded counts/minute. Participants wore the accelerometers on their right hip (above the iliac crest), secured with an elastic belt [24]. The first six participants wore only one Actical during the protocol, which resulted in some missing accelerometry data due to malfunctioning equipment. For the subsequent participants, three accelerometers were used to protect against data loss and to provide a measure of reliability across devices.

### Procedure

Data were collected from each participant in one visit. Trained and certified measurement staff completed a standardized protocol with each participant. After completing informed consent, participants were measured for height and weight and completed the brief demographic questionnaire. Accelerometer counts/minute and walking lap-times were collected as participants completed the five prescribed tasks. Rest periods were allowed between activities.

Five activities were completed in either an open-floor gymnasium or a large recreation room. In each location, the research team set up the measurement station which included a table and chairs and a designated “walking track” using masking tape and athletic cones. The tape and/or cones marked the starting line and the turn-around point 50 feet from the starting line. Using scripted, standardized instructions, research staff instructed the participants to perform five activities for five minutes each: one seated task (card sorting), one standing task (table dusting), and walking under three sets of instructions: “strolling”; “walking for exercise”; and “walking in an emergency.” These five tasks were chosen to capture a range of physical activities from sedentary to vigorous, and the three types of walking instructions were intended to capture a range of self-selected walking paces: a slow leisurely walking pace, a moderate walking pace typical of exercise, and the fastest walking pace possible. Using a laptop computer which was synchronized to the accelerometers, the research staff began each task at the start of a minute so that the 5 epochs of recorded accelerometry data would correspond directly to the 5 minutes of activity for each task. The start time for each task was recorded to retrieve corresponding data from the Actical software.

The first task for each participant was card sorting. The participant sat at a table and, when told to begin, shuffled the deck of cards three times then sorted by suit, then sorted by number, until the 5 minutes were complete. Next, all participants were asked to stand at the same table. When told to begin, they used a small feather duster to dust the table and three picture frames. Participants were instructed to “please dust the table and the items like you would normally do at home” and continued to dust the table and items for 5 minutes. Next, the participants completed the walking tasks; the order of walking instructions was counter-balanced such that each participant completed the three walking tasks in a random order. The walking instructions were delivered from a script to ensure consistency across measurement staff. The strolling instructions were: “Walk like you are browsing the aisles of the grocery store or are window shopping at the mall.” The “walking for exercise” instructions were: “walk like you are beginning a 30 minute walk for exercise.” The “walking in an emergency” instructions were: “walk like you are in a hurry to get to a loved one who is in need.” For each walking pace, the participant stood at the starting line of the “walking track”, listened to the instructions, and when told to begin, walked down and back on the walking track for 5 minutes. Using a stopwatch, the measurement staff timed and recorded the time for each lap (down and back on the track) as well as the total number of laps walked (to the nearest inch). The lap-time data enabled the researchers to calculate the average pace in miles per hour for each walking task.

### Statistical analyses

Statistical analyses were conducted in the statistical package R (Version 2.13.1). Data from five participants were incomplete (due to equipment malfunction or failure to complete the study protocol) and, therefore, dropped from analyses to maintain the integrity of the data. Likewise, four accelerometers malfunctioned and recorded data that were out of the acceptable range, and the data from these devices were dropped universally across participants. Therefore, analyses included one to three Acticals per participant. To test for reliability across the three Acticals, intra-class correlations were calculated for each task (devices nested within individual) using unconditional multilevel models. High intra-class correlations (> .94) indicated good reliability and showed that less than 6% of the variance in activity estimates was due to differences in devices or measurement error. Therefore, the average counts/minute across each participant’s Acticals was used in further analyses.

In these analyses, walking pace was used as an estimate of PA intensity. Preliminary visual examination of the data confirmed that the card sorting task and the table dusting task were distinguishable from the locomotive activities. The card sorting task and the table dusting task had no measure of intensity (no calculated pace) and were not included in subsequent analyses given the primary focus on understanding self-selected walking paces. The average walking pace was calculated for each walking task from lap-time data for minutes 2–4 of each activity.

**Figure 1:**
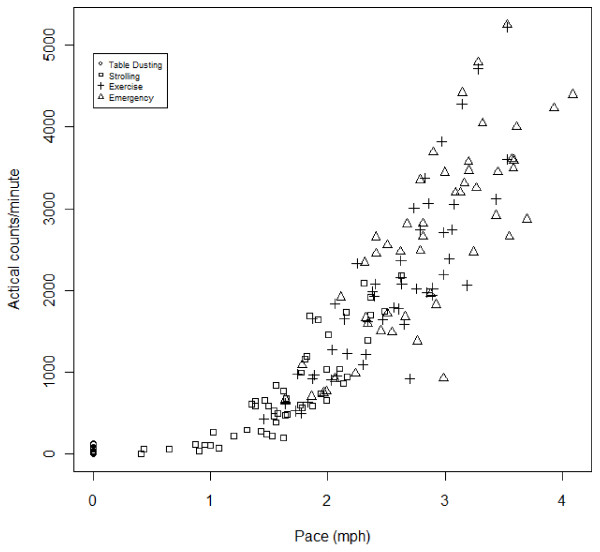
Walking pace vs. Actical counts/minute (Note: Table dusting was assigned a pace of 0.0 mph.).

After reviewing the distributions of walking paces for each activity 2.0 mph was chosen as the pace that differentiated walking for exercise (moderate PA) from strolling. Specifically, 67% of the sample’s self-selected “walking for exercise” pace was greater than 2.0 mph, while less than 33% of the sample’s self-selected “strolling” pace was greater than 2.0 mph. Furthermore, 2.0 mph was similar to, but slightly lower than, the walking paces associated with moderate PA used in previous Actical calibration studies [10,12]. A regression analysis was used to find the Actical counts/minute corresponding to moderate PA (walking at 2.0 mph). Actical counts/minute were plotted against walking paces (see Figure 1). Actical counts/minute and walking pace demonstrated a curvilinear relationship. More specifically, Actical counts/minute increased more rapidly as walking pace increased, such that going from 1.0 to 2.0 mph was associated with a smaller increase in counts while going from 3.0 to 4.0 mph was associated with a larger increase in counts. Therefore, a square root transformation was used for Actical counts/minute to adjust for this curvilinear relationship. Transformed Actical counts/minute was regressed onto walking pace. Using Equation 1, the Actical counts/minute at 2.0 mph was calculated as the cut point for moderate PA.

## Results and discussion

### Participant characteristics

The participant characteristics are presented in Table 1. They were 61% female, and the average age was 60.1 years (SD = 9.9). The average BMI was 30.5 kg/m^2^ (SD = 6.0).

**Table 1:** Participant characteristics (N = 51)

***Demographics and Health Characteristics***	N (%)*
Sex	
Female	31 (61)
Male	20 (39)
Age, years, Mean (SD)	60.1 (9.9)
BMI, kg/m^2^, Mean (SD)	30.5 (6.0)
Income	
<$10,000	8 (16)
$10,000-24,000	7 (14)
>$25,000	33 (65)
Education	
< HS Degree	3 (6)
HS Degree	14 (28)
> HS Degree	32 (63)
Employment	
Working	19 (37)
Retired/Permanently Disabled	25 (49)
Other	7 (14)
Marital Status	
Married/In an Unmarried Couple	20 (39)
Widowed/Separated/Divorced	24 (47)
Never Married	7 (14)
*Walking Patterns*	
Walks for Pleasure	
Yes	15 (29)
No	36 (71)
Minutes/Day Walking for Pleasure, Mean (SD)	13.1 (11.4)

BMI = Body Mass Index; SD = Standard Deviation.

*Except for Age, BMI, and Minutes/Day Walking for Pleasure which are reported as Mean (SD).

### Accelerometer counts and walking paces

Table 2 presents the Actical counts/minute and paces for the five physical activity tasks. As expected, Actical counts/minute increased with the intensity of the activity, indicating that the protocol and standardized instructions elicited physical activity of the desired intensities. The average paces for “strolling”, “walking for exercise”, and “walking in an emergency” were 1.62 mph (SD = .51), 2.51 mph (SD = .53), and 2.86 mph (SD = .58), respectively. Self-selected walking paces increased as expected.

**Table 2:** Average pace (mph) and Actical counts/minute for each activity

	**Average pace (SD)**	**Average Actical counts/minute (SD)**
Card Sorting	--	4 (15)
Table Dusting	--	16 (29)
“Strolling”	1.62 (0.51)	751 (591)
“Walking for Exercise”	2.51 (0.53)	2006 (1095)
“Walking in Emergency”	2.86 (0.58)	2617 (1169)

SD = Standard Deviation.

### Moderate PA threshold

**Figure 2:**
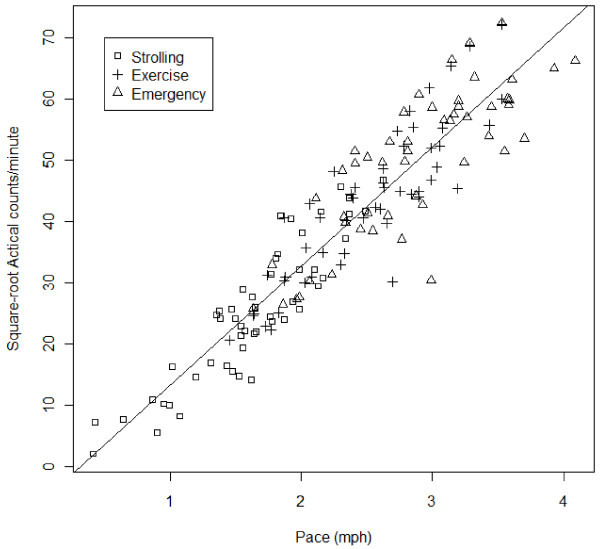
Walking pace vs. square root transformed Actical counts/minute.

Figure 1 shows the relationship between walking pace and Actical counts/minute. Figure 2 shows walking pace versus square root transformed Actical counts/minute. The model was statistically significant, (F(1,151) = 886.9, p < .001, R^2^ = .85). The best fit line for the regression of square-root of Actical counts/minute onto pace was: **Equation 1:**$\surd \text{counts}/\text{minute}=-6.\text{2105}+\text{19}.\text{5001}\left(\text{pace}\right)$.

Using Equation 1, the cut point for moderate PA was calculated to be 1075 counts/minute (SEM = 73 counts/minute).

## Discussion

In this study, we identified an intensity-specific cut point for use with the Actical accelerometer for moderate PA with older, African American adults. Unlike the Actigraph, Actical accelerometers are relatively new to the field of PA measurement, and there is a paucity of calibration and validation studies for this device in adults [25]. The present study resulted in a cut point of 1075 counts/minute (SEM = 73 counts/minute), which is similar to the cut point of 1065 counts/minute established by Hooker and colleagues in an older adult sample [13]. Thus, these results corroborate the existing cut points for older adults, validating the use of adjusted cut point for older populations, and extend the generalizability of the adjusted cut points to African American populations. Moreover, these results complement the existing literature by utilizing a novel protocol and a definition of moderate PA relative to the participants’ subjective experience of intensity versus more objectively defined moderate PA. A recent review of accelerometer calibration studies suggested that “[a] combination of laboratory- and field-based studies may enhance the appropriate calibration of accelerometer output to the underlying activity patterns in the target population” [25]. This study, then, meets current recommendations for well-established accelerometer cut points and strengthens our confidence in the use of the modified cut point for an ethnically diverse, older adult population.

Given that older adults engage in different types and intensities of PA than younger adults and children [11], it is important to develop modified cut points for a given population. Modified cut points are necessary because using cut points established in younger, healthier populations may lead researchers to miss meaningful physical activity changes in the context of health promotion interventions. The new cut point enables researchers to assess moderate PA more accurately for individuals who are older and who may have chronic illness which likely may lower the absolute intensity of moderate PA. A growing body of literature recognizes that relative PA intensity may vary by age-related differences in fitness with older individuals reaching higher energy expenditure and heart health benefits at lower intensity levels than younger and healthier individuals [14,15,17,26]. Experts in the field of PA measurement have recognized that existing guidelines established in young, healthy adults should be adjusted for underserved and minority populations who are older and in poor health [2,11,17,18], since age-related decreases in resting metabolic rate may cause errors in EE calculations that were developed in younger samples [11].

The self-selected walking paces observed in the present study were, in fact, similar to previously published figures in studies with older adults [14-16]. Moreover, the self-selected pace for “walking for exercise”, was slightly lower than those observed in previous Actical cut point studies using younger, healthier participants [10,12]. Thus, the cut point associated with “walking for exercise”, a subjective determinant of moderate PA, in an underserved older, African American adult population, was lower than those associated with moderate PA in a younger, normal weight population.

In summary, the cut point for older adults allows researchers to more accurately demonstrate efficacy and potentially meaningful changes in PA for older minority populations, which are known to have lower levels of PA [19-22]. The current study provided cut points for moderate PA that were more appropriate for our on-going Positive Action for Today’s Health (PATH) trial [27]. As such, this paper presents a model for developing cut points tailored to the unique health characteristics of an underserved population of interest. By comparing Actical counts to a range of PA activities and by defining moderate PA as “walking for exercise,” an activity that was appropriate for our population, this novel calibration approach defined the cut point relative to the moderate PA intensity determined by the participants’ subjective experience. By including activities at the low end of the activity intensity spectrum and by targeting a specific type of activity behavior, this methodology is in line with current recommendations in the field of accelerometry calibration [25]. Furthermore, unlike protocols that require measures of oxygen uptake in a control laboratory environment, the current study provides an easily replicable protocol for other researchers interested in exploring the validity of accelerometer cut points in special populations.

The results presented here must be considered in light of the study’s limitations. This study had a relatively small sample size. Future research should aim for a larger sample size to enable sub-group analyses, when necessary, and to improve the precision and power of their statistical analyses in general. Future studies would benefit from including an informal assessment of PA intensity, such as the “talk but not sing” test described in the US DHHS PA Guidelines [2] or the Borg’s Rating of Perceived Exertion [28]. These measures would enable others to confirm that their population is reaching relative moderate intensity PA when “walking for exercise.” Similarly, a subjective rating of PA intensity would allow researchers to feel more confident that the different instructions for walking resulted in subjectively different intensity levels.

## Conclusions

This study provides a moderate intensity PA cut point that is now available for use with older, African American adults. This study also provides a methodology for identifying a new accelerometry cut point for researchers and interventionists working with unique populations. The implications of this work suggest that future investigators need to integrate these types of methodologies to validate existing cut points or determine specific cut point parameters for accelerometry data when appropriate. This will enable researchers to more accurately estimate PA and promote PA changes that match the ability of in their population of interest.

## Abbreviations

SD: Standard deviation; kg/m2: Kilograms per square-meter; SEM: Standard error of measure.

## Competing interests

The authors declare that they have no competing interests.

## Authors’ contributions

NNT contributed to the conception and design of the study, oversaw the acquisition of data, conducted a portion of the analyses, and drafted the manuscript. HGL performed the majority of the statistical analyses and helped to draft the manuscript. DKW participated in the design of the study and helped to draft the manuscript. RRP participated in the study design and conception. LVH participated in the design of the study and statistical analyses. AKT participated in the acquisition of data and helped draft the manuscript. All authors read and approved the final manuscript.
